# A 54‐Year‐Old Male With Persistent Air Leak After Endotracheal Intubation

**DOI:** 10.1002/rcr2.70591

**Published:** 2026-04-22

**Authors:** Angela Montes, Rodrigo Garcia Tome, William West, Brett Lindgren, Patrick Chan

**Affiliations:** ^1^ Department of Internal Medicine University of Southern California Los Angeles California USA; ^2^ Division of Pulmonary and Critical Care Medicine, Department of Medicine, Los Angeles General Medical Center University of Southern California Los Angeles California USA; ^3^ Division of Pulmonary and Critical Care Medicine, Department of Medicine University of Southern California Los Angeles California USA

**Keywords:** bronchoscopy, cuff leak, endotracheal intubation, persistent air leak, tracheoesophageal fistula

## Abstract

Tracheoesophageal fistulas (TEFs) are abnormal communications between the trachea and oesophagus that may be congenital or acquired. Clinical presentation can be subtle and nonspecific. We report a case of a 54‐year‐old male with advanced HIV, end‐stage renal disease and multiple comorbidities who developed a persistent air leak after emergent intubation following cardiac arrest. Despite endotracheal tube exchange, the air leak persisted and was accompanied by marked abdominal distension. Computed tomography (CT) imaging revealed a tracheal defect near the left mainstem bronchus and bronchoscopy confirmed a TEF. The patient underwent oesophageal stent placement; however, methylene blue testing demonstrated a persistent communication between the oesophagus and airway. Given his poor overall prognosis, the family opted to withdraw life‐sustaining treatment. This case highlights the importance of considering TEF in mechanically ventilated patients with persistent air leak unresponsive to tube exchange.

## Graphical abstract

A 54‐year‐old male developed a persistent air leak following emergent intubation after cardiac arrest. Imaging and bronchoscopy revealed a tracheoesophageal fistula, which persisted despite oesophageal stent placement. This case underscores the importance of considering tracheoesophageal fistula in mechanically ventilated patients with unexplained persistent air leaks and abdominal distension.

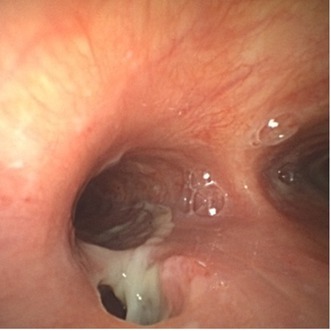

## 1 Introduction

Tracheoesophageal fistulas (TEFs) are abnormal communications between the trachea and oesophagus that may be congenital or acquired. Acquired TEFs are further classified as benign or malignant. Benign TEFs may result from prolonged mechanical ventilation, excessive endotracheal tube cuff pressure, trauma, surgery or infection, while malignant TEFs typically arise from locally invasive cancers of the oesophagus, trachea, lung, thyroid or larynx [1]. Clinical presentation can be subtle and nonspecific. In mechanically ventilated patients, TEF may manifest as a persistent air leak, difficulty maintaining ventilator volumes or abdominal distension. Early recognition is crucial to not delay management.

## 2 Case Report

A 54‐year‐old male with a history of HIV (CD4 count 98), coronary artery disease, end‐stage renal disease on haemodialysis, hypertension and diabetes mellitus presented with 2 days of nausea, vomiting and subjective fevers. He was admitted to the medical intensive care unit (MICU) for mixed cardiogenic and septic shock due to *Myroides* bacteraemia and later transferred to the floor after improvement.

Several days later, he developed progressive abdominal bloating and frequent belching. He then developed acute hypoxic respiratory failure suspected secondary to aspiration and was readmitted to the MICU. Flexible endoscopic evaluation of swallowing (FEES) by speech‐language pathology demonstrated aspiration across all consistencies. Shortly after the procedure, the patient went into pulseless electrical activity (PEA) arrest. Return of spontaneous circulation (ROSC) was achieved after 7 min, and he was emergently intubated.

**FIGURE 1:**
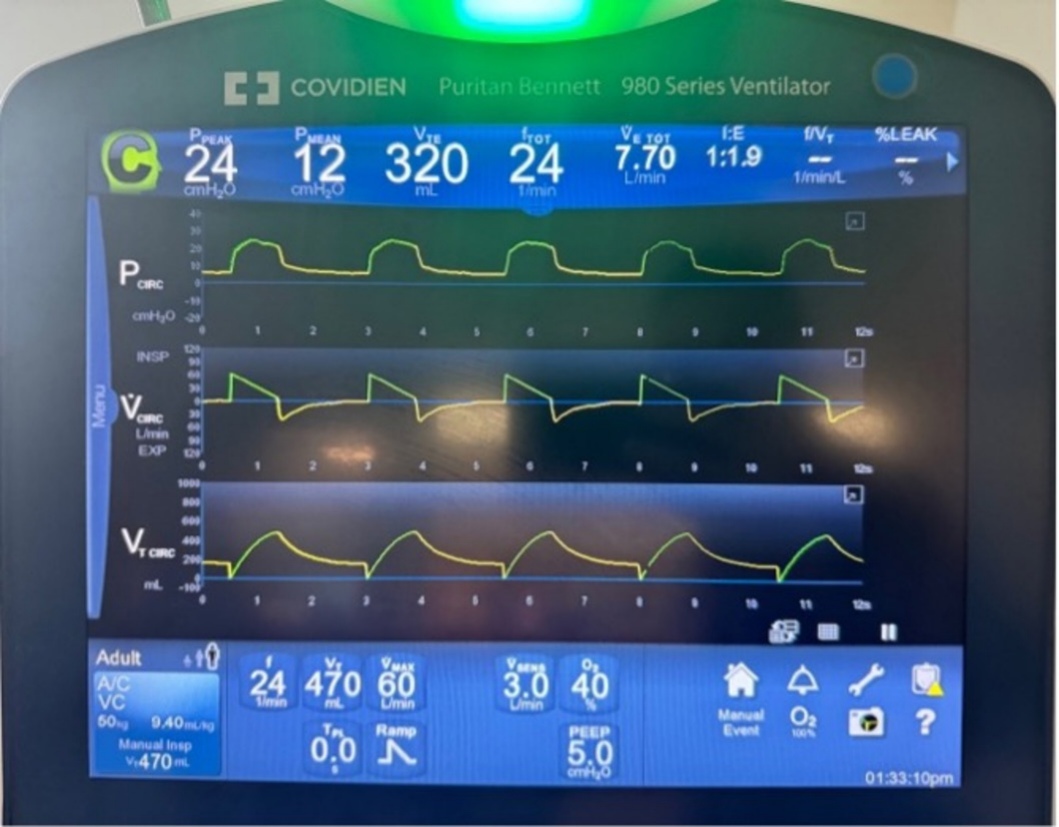
Ventilator waveforms demonstrating volume‐time curve not returning to baseline on expiration and notable difference in inspiratory and expiratory tidal volumes, suspicious for an air leak.

The pilot balloon was noted to be deflated despite multiple attempts at reinflation, raising concern for ruptured cuff. The endotracheal tube (ETT) was exchanged, but an audible air leak was present (Video 1). A nasogastric tube was in place and connected to wall suction, and the abdomen was noted to be distended. Ventilator waveforms showed a notable difference between inspiratory and expiratory volumes (Figure 1).

**VIDEO 1:** Audible air leak from the endotracheal tube despite adequate cuff inflation and confirmed tracheal positioning. Video content can be viewed at https://onlinelibrary.wiley.com/doi/10.1002/rcr2.70591.

**FIGURE 2:**
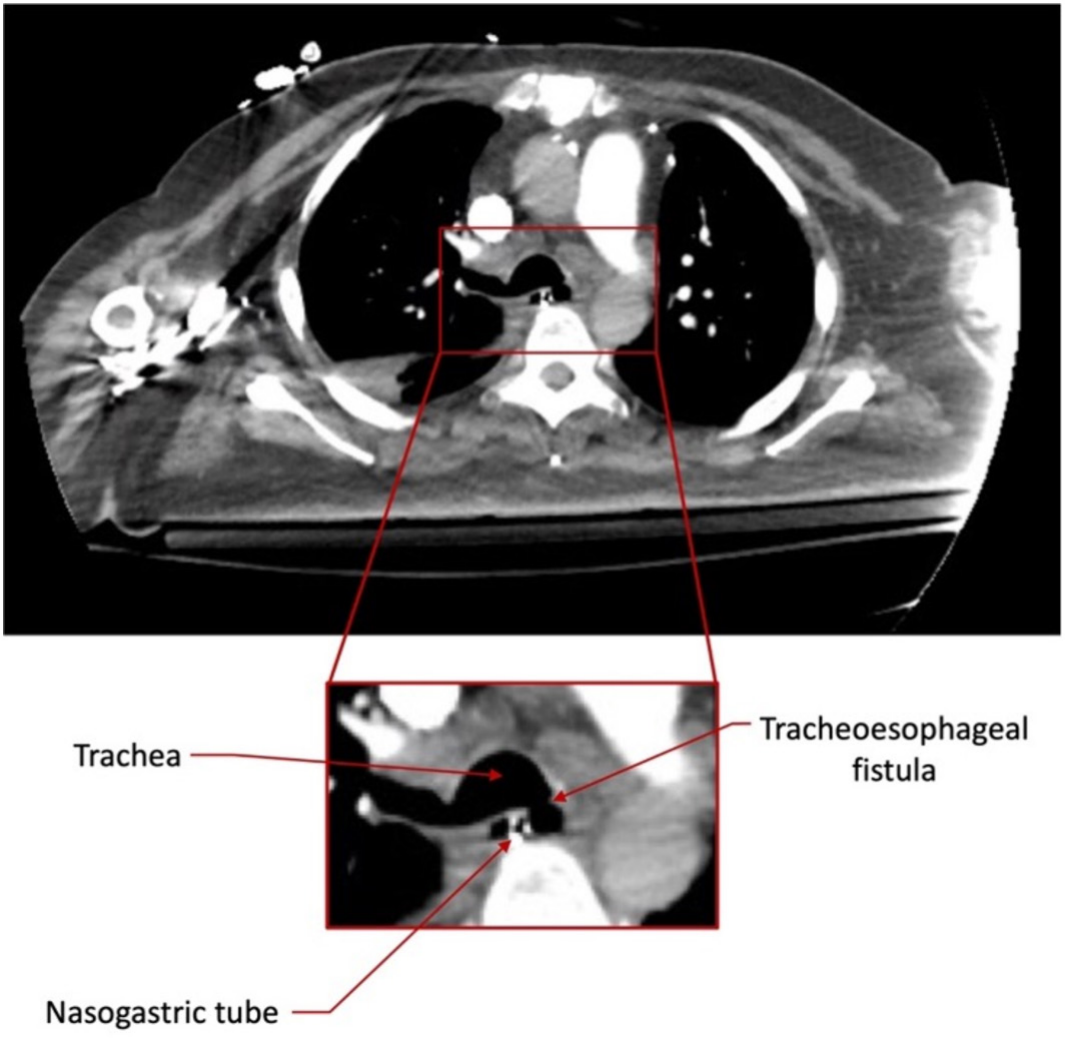
Computed tomography (CT) scan of the chest demonstrating a tracheal defect at the entrance of the left mainstem bronchus.

**FIGURE 3:**
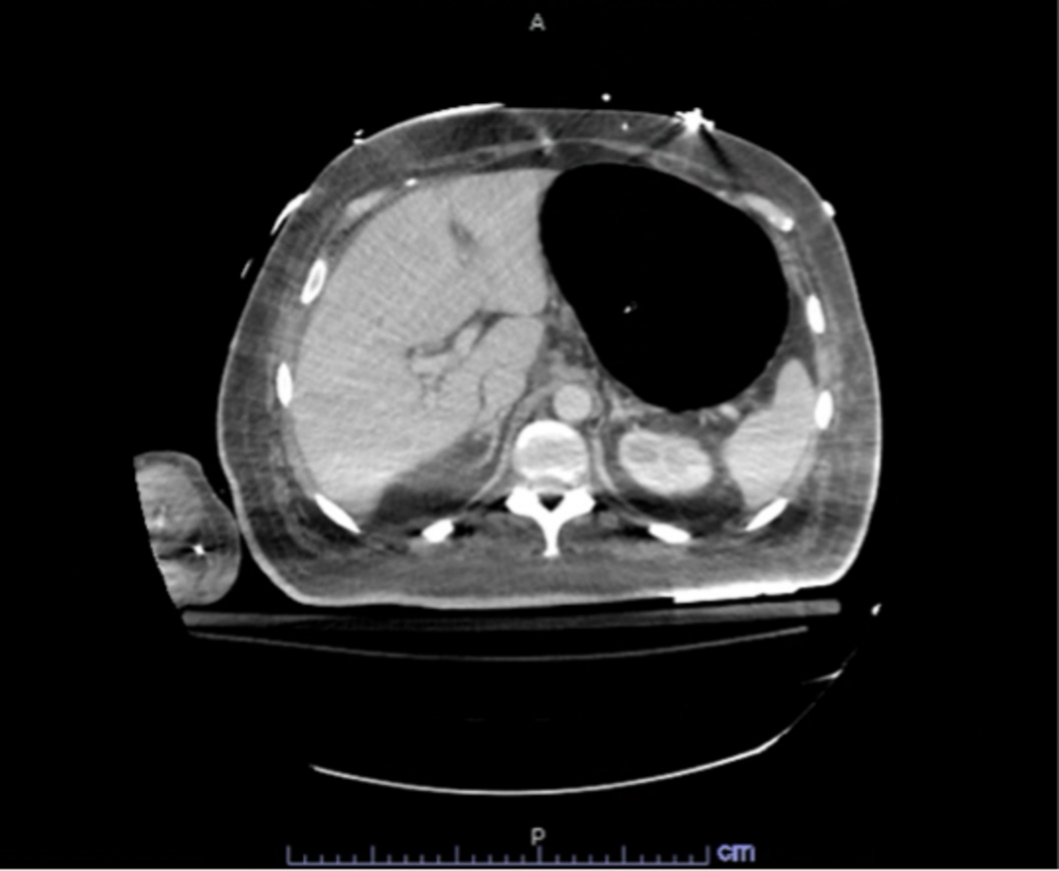
Computed tomography (CT) scan of the abdomen showing marked gaseous distension of the stomach.

**FIGURE 4:**
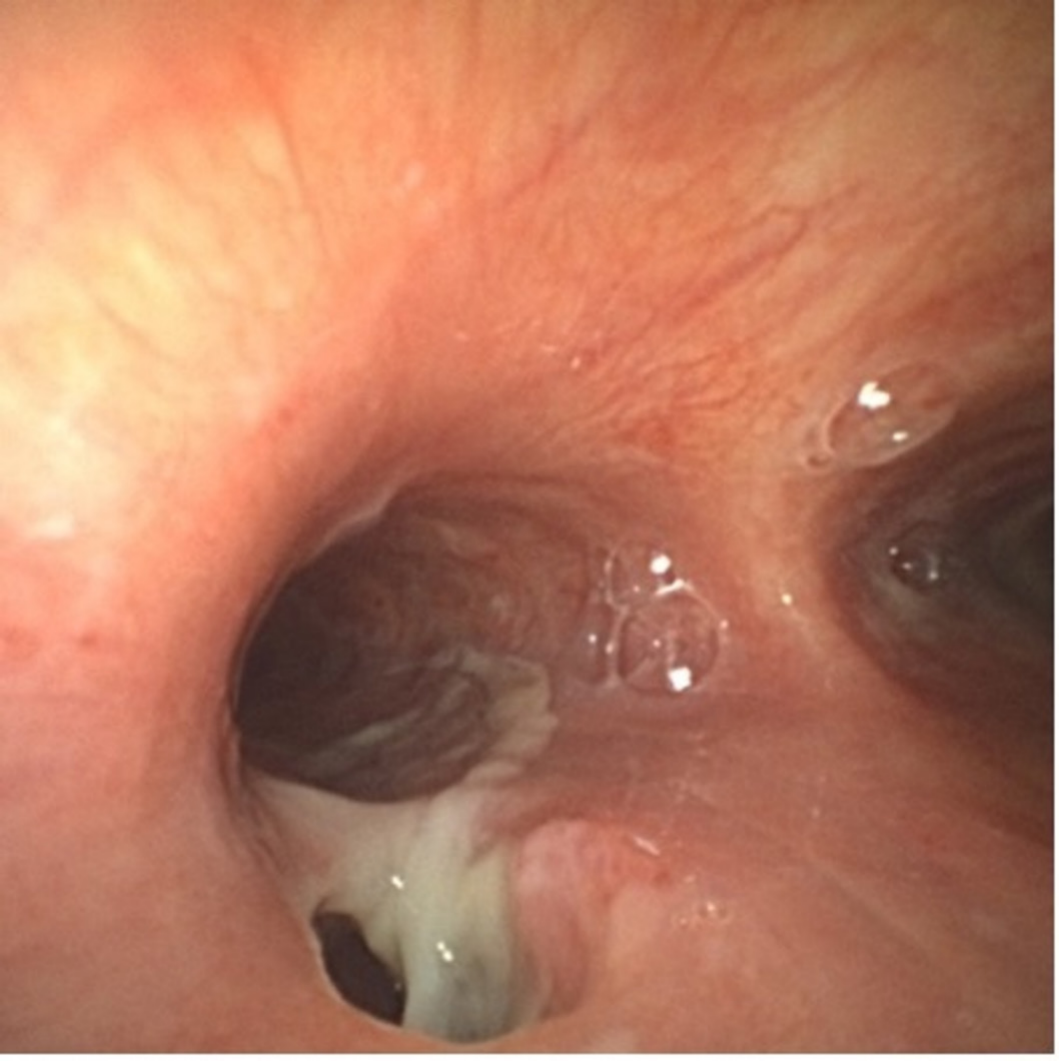
Bronchoscopic visualization of a tracheal defect adjacent to the left mainstem bronchus.

Initial bedside bronchoscopy through the ETT to the distal trachea demonstrated appropriate positioning of the tube. Arterial blood gas showed adequate oxygenation and ventilation. CT of the chest and abdomen revealed a tracheal defect near the left mainstem bronchus (Figure 2) with marked gaseous distension of the stomach (Figure 3). A repeat bronchoscopy confirmed the presence of the tracheal defect (Figure 4).

**FIGURE 5:**
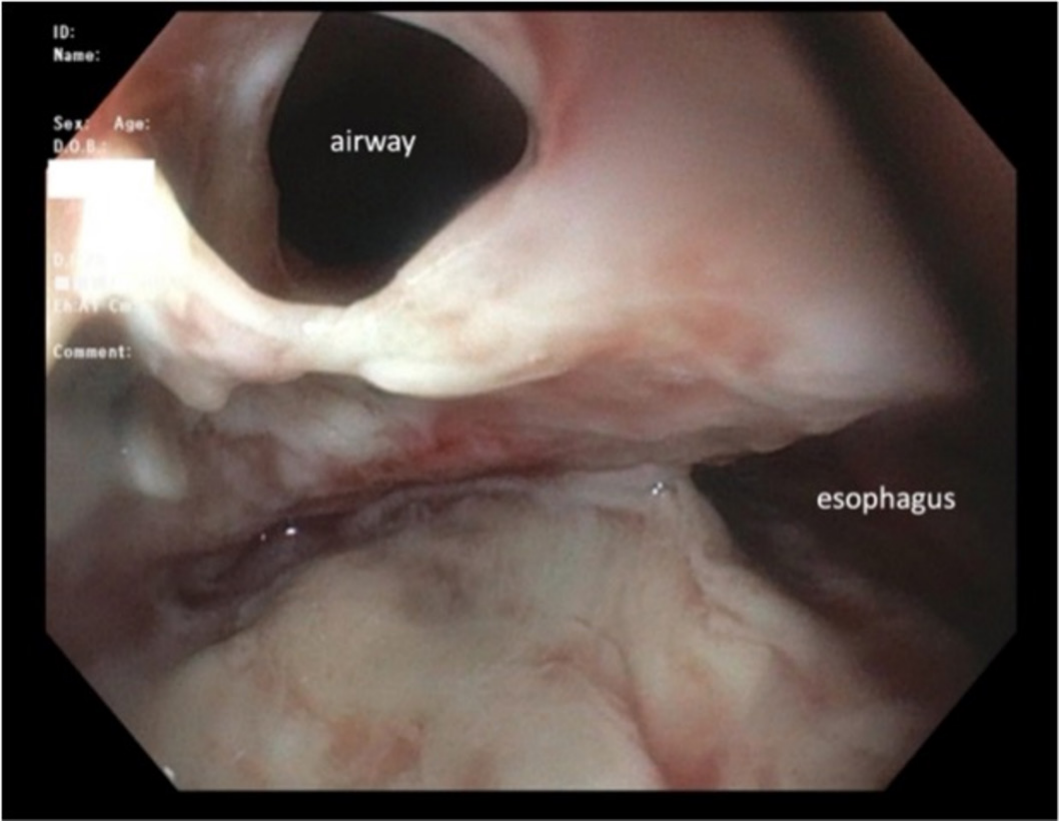
Esophagogastroduodenoscopic view showing direct visualization of trachea through oesophageal defect.

After bronchoscopy confirmed a TEF at the entrance of the left mainstem bronchus, thoracic surgery was consulted. Given the fistula's chronicity and proximity to carina, surgical repair was deferred in favour of endoscopic management. The patient underwent esophagogastroduodenoscopy (EGD) (Figure 5) and bronchoscopy together for placement of oesophageal stent and simultaneous placement of percutaneous endoscopy gastrostomy (PEG). Although a persistent gap between the oesophageal wall and the stent was noted (Video 2), the team felt adequate sealing would occur as oesophageal stents typically expand over 24–48 h.

**VIDEO 2:** Bronchoscopic visualization of residual gap between the oesophageal wall and the stent shortly after stent placement. Video content can be viewed at https://onlinelibrary.wiley.com/doi/10.1002/rcr2.70591.

After the procedure, the patient's abdomen remained distended. Opening the PEG to air revealed a large air leak synchronous with ventilator breaths (Video 3). A repeat bronchoscopy was performed due to concern for persistent fistula and visualized thick, white bilateral lower lobe secretions. A leak test was performed—methylene blue instilled into the PEG showed no airway leakage. However, methylene blue instillation into the oesophagus proximal to the stent resulted in immediate airway soiling (Video 4), confirming a persistent fistula despite oesophageal stent placement. After extensive discussion, the family elected to withdraw life‐sustaining treatment given poor prognosis and need for further intervention. The patient passed away shortly after compassionate extubation.

**VIDEO 3:** PEG tube opening demonstrates a significant air leak synchronous with ventilator breaths, suggesting ongoing communication between the trachea and oesophagus despite oesophageal stent placement. Video content can be viewed at https://onlinelibrary.wiley.com/doi/10.1002/rcr2.70591.

**VIDEO 4:** Bronchoscopic visualization of methylene blue entering the airway following instillation into the oesophagus proximal to the stent, confirming persistent tracheoesophageal fistula. Video content can be viewed at https://onlinelibrary.wiley.com/doi/10.1002/rcr2.70591.

## 3 Discussion

TEFs may present with nonspecific clinical manifestations that vary by size and location. Cough associated with swallowing (Ono's sign) is classically described; other common symptoms include fevers, dysphagia and chest pain [2]. In mechanically ventilated patients, persistent air leak is commonly attributed to endotracheal tube‐associated issues such as inadequate cuff inflation, cuff rupture, or tube malposition. When an air leak persists despite ETT exchange and confirmation of appropriate tracheal placement, non‐endotracheal tube‐related causes such as TEF should be considered. Although abdominal distension is classically associated with oesophageal intubation, its presence with a persistent air leak should raise suspicion for TEF.

TEF can be diagnosed using contrast oesophagram, computed tomography (CT), bronchoscopy, or endoscopy. CT oesophagram has the highest sensitivity and is preferred when patients can tolerate oral contrast. In patients unable to swallow, CT scan becomes the diagnostic modality of choice [3]. Although bronchoscopy is readily feasible in intubated patients, its diagnostic yield for TEF is limited by relatively low sensitivity. It requires careful inspection of the entire trachea and segmental levels. TEFs may be hidden behind tracheal rings or obscured during dynamic changes during respiration [4]. Techniques to improve visualization of the fistula include using oxygen insufflation or methylene blue [1].

Management of TEFs depends on aetiology and patient condition. Surgical management is preferred for benign TEFs when feasible, though endoscopic stent placement has been shown to be a safe alternative [5]. Oesophageal stents are usually chosen over airway stents as oesophageal stents are generally well‐tolerated. Dual oesophageal and airway stents may be required in complex cases but carry a risk of expanding forces exacerbating local tissue ischemia and necrosis [1].

Oesophageal biopsies were negative for malignancy in this patient, supporting a diagnosis of benign TEF. His immunocompromised state, reflected by advanced HIV, ESRD and diabetes, placed him at high risk for opportunistic infections, leading to oesophageal inflammation and ulceration contributing to fistula formation. In retrospect, this patient likely had a clinically occult TEF until precipitation by aspiration or mechanical ventilation. This case highlights the importance of considering TEF in the differential diagnosis of a persistent air leak unresponsive to endotracheal tube exchange.

## Author Contributions

Angela Montes and Patrick Chan drafted the initial manuscript, and Rodrigo Garcia Tome prepared the media. All authors revised the manuscript and approved the final version of the manuscript.

## Funding

The authors have nothing to report.

## Consent

The authors declare that written informed consent was obtained for the publication of this manuscript and accompanying images and attest that the form used to obtain consent from the patient complies with the Journal requirements as outlined in the author guidelines.

## Conflicts of Interest

The authors declare no conflicts of interest.

## Data Availability

The data that support the findings of this study are available from the corresponding author upon reasonable request.
